# FUCOIDANS ARE NOVEL SENOTHERAPEUTICS THAT ENHANCE SIRT6 AND DNA REPAIR ACTIVITY

**DOI:** 10.1093/geroni/igac059.2666

**Published:** 2022-12-20

**Authors:** Lei Zhang, Brian Hughes, Gregory Tombline, Luise Angelini, Matt Yousefzadeh, Vera Gorbunova, Laura Niedernhofer, Paul Robbins

**Affiliations:** University of Minnesota, Saint Paul, Minnesota, United States; University of Minnesota, Minneapolis, Minnesota, United States; University of Rochester, Rochester, New York, United States; University of Minnesota, Minneapolis, Minnesota, United States; University of Minnesota, Minneapolis, Minnesota, United States; University of Rochester, Rochester, New York, United States; University of Minnesota, Minneapolis, Minnesota, United States; University of Minnesota, Minneapolis, Minnesota, United States

## Abstract

With age, senescent cells accumulate in various tissues where they contribute to loss of tissue homeostasis, aging, and age-related diseases through their inflammatory senescence-associated secretory phenotypes (SASPs). Senotherapeutics able to selectively eliminate senescent cells, termed senolytics, or suppress the detrimental SASPs, termed senomorphics, have been demonstrated to improve age-associated comorbidities and aging phenotypes. To discover novel senotherapeutics translatable to promote healthy longevity, we conducted a drug screening of diverse natural products based on the characteristic senescence-associated β-galactosidase activity. Several fucoidans from different brown seaweed were found to exhibit potent senotherapeutic activity. Fucoidans are long-chain sulfated polysaccharides found in various species of brown algae including seaweed. The best senomorphic fucoidan was able to suppress senescence in cultured senescent fibroblasts, in ex vivo human tissue explants, and in vivo in mouse models of natural and accelerated aging. Specifically, fucoidan reduced markers of cellular senescence and SASP in senescent mouse and human cells. Acute treatment of the fucoidan in naturally aged mice reduced tissue senescence, especially in the kidney and lung. Chronic treatment of the fucoidan in Ercc1-/Δ progeria mice attenuated composite aging symptoms and extended healthspan. Interestingly, preliminary mechanistic studies demonstrated that fucoidan can improve non-homologous end-joining-directed DNA damage repair and increase the mono-ADP-ribosylation activity of SIRT6, suggesting a relationship between cellular senescence, DNA repair, and SIRT6 signaling pathways. Collectively, fucoidans were identified as novel senotherapeutics with translational potential for reducing cellular senescence, ameliorating age-associated phenotypes, and extending healthspan as well as able improve DNA repair pathways through modulation of SIRT6 activity.

